# ACTIVATE-2: A Double-Blind Randomized Trial of BCG Vaccination Against COVID-19 in Individuals at Risk

**DOI:** 10.3389/fimmu.2022.873067

**Published:** 2022-07-05

**Authors:** Maria Tsilika, Esther Taks, Konstantinos Dolianitis, Antigone Kotsaki, Konstantinos Leventogiannis, Christina Damoulari, Maria Kostoula, Maria Paneta, Georgios Adamis, Ilias Papanikolaou, Kimon Stamatelopoulos, Amalia Bolanou, Konstantinos Katsaros, Christina Delavinia, Ioannis Perdios, Aggeliki Pandi, Konstantinos Tsiakos, Nektarios Proios, Emmanouela Kalogianni, Ioannis Delis, Efstathios Skliros, Karolina Akinosoglou, Aggeliki Perdikouli, Garyfallia Poulakou, Haralampos Milionis, Eva Athanassopoulou, Eleftheria Kalpaki, Leda Efstratiou, Varvara Perraki, Antonios Papadopoulos, Mihai G. Netea, Evangelos J. Giamarellos-Bourboulis

**Affiliations:** ^1^ 4^th^Department of Internal Medicine, National and Kapodistrian University of Athens, Medical School, Athens, Greece; ^2^ Department of Internal Medicine and Center for Infectious Diseases, Radboud University, Nijmegen, Netherlands; ^3^ Department of Internal Medicine, “Bodosakeio” General Hospital of Ptolemaida, Ptolemaida, Greece; ^4^ 1^st^Department of Internal Medicine, “G.Gennimatas” Athens General Hospital, Athens, Greece; ^5^ Department of Pulmonary Medicine, Aghia Eirini General Hospital of Kerkyra, Kontokali, Greece; ^6^ Department of Therapeutics, National and Kapodistrian University of Athens, Athens, Greece; ^7^ Department of Surgery, General Hospital of Argos-Unit of Nafplion, Nafplion, Greece; ^8^ 3^rd^Department of Internal Medicine, National and Kapodistrian University of Athens, Athens, Greece; ^9^ Department of Internal Medicine, General Hospital of Karditsa, Karditsa, Greece; ^10^ Nemea Health Center, Nemea, Greece; ^11^ Department of Internal Medicine, Patras University Hospital, Rio, Greece; ^12^ 1^st^Department of Internal Medicine, University Hospital of Ioannina, Ioannina, Greece; ^13^ Hellenic Institute for the Study of Sepsis, Athens, Greece; ^14^ Department of Immunology and Metabolism, Life and Medical Sciences Institute, University of Bonn, Bonn, Germany

**Keywords:** BCG, COVID-19, SARS-CoV-2, elderly vaccination, trained immunity

## Abstract

In a recent study of our group with the acronym ACTIVATE, Bacillus Calmete-Guérin (BCG) vaccination reduced the occurrence of new infections compared to placebo vaccination in the elderly. Most benefit was found for respiratory infections. The ACTIVATE-2 study was launched to assess the efficacy of BCG vaccination against coronavirus disease 2019 (COVID-19). In this multicenter, double-blind trial, 301 volunteers aged 50 years or older were randomized (1:1) to be vaccinated with BCG or placebo. The trial end points were the incidence of COVID-19 and the presence of anti–severe acute respiratory syndrome coronavirus 2 (anti–SARS-CoV-2) antibodies, which were both evaluated through 6 months after study intervention. Results revealed 68% relative reduction of the risk to develop COVID-19, using clinical criteria or/and laboratory diagnosis, in the group of BCG vaccine recipients compared with placebo-vaccinated controls, during a 6-month follow-up (OR 0.32, 95% CI 0.13-0.79). In total, eight patients were in need of hospitalization for COVID-19: six in the placebo group and two in the BCG group. Three months after study intervention, positive anti–SARS-CoV-2 antibodies were noted in 1.3% of volunteers in the placebo group and in 4.7% of participants in BCG-vaccinated group. The ACTIVATE II trial did not meet the primary endpoint of the reduction of the risk for COVID-19 3 months after BCG vaccination; however, the secondary endpoint of the reduction of the risk for COVID-19 6 months after BCG vaccination was met. BCG vaccination may be a promising approach against the COVID-19 pandemic.

## Introduction

At the end of 2019, a novel Betacoronavirus emerged in Wuhan, China, and in February 2020, the virus was named severe acute respiratory syndrome coronavirus 2 (SARS-CoV-2), and it is responsible for coronavirus disease 2019 (COVID-19) (1). Since the beginning of 2020, the virus rapidly spread worldwide and it has had devastating consequences globally, causing a pandemic that brought suffering and death to millions of people.

The most important preventive tool against a new infection are vaccines, and a concerted effort has been launched around the world for the development of safe and effective vaccines against COVID-19. As the beginning of 2021, a mere 1 year later, several new and effective vaccines against this new infection have been developed, based on both old (inactivated virus and recombinant proteins) and new (mRNA technology and adenovirus vectors) platform technologies (2). Vaccination programs with several of these vaccines have been initiated, and the beneficial impact of vaccination on the pandemic in countries that managed to vaccinate a significant proportion of the population is already starting to be felt (3). Unfortunately, the design and development of successful specific vaccines are time-consuming, with shortages of vaccines being encountered in the majority of countries: it is expected that 2–3 years will be needed until vaccines will be sufficiently available to all countries.

These inherent challenges posed by the development of completely new vaccines against a new pathogen argue for the identification of alternative preventive approaches that could at least partially protect the population until specific vaccines are developed and become available worldwide. One such approach that has been proposed for the protection against COVID-19 is the use of live-attenuated vaccines with known protective effects against heterologous infections (4). Epidemiological findings have suggested that Bacillus Calmette-Guérin (BCG), which is a live attenuated vaccine and is currently used to prevent tuberculosis, can also generate positive effect against other infectious diseases (5). In neonates, BCG vaccination at birth decreased child mortality, and this effect was attributed to a lower incidence of neonatal sepsis and respiratory infections (6–8), whereas a 70% reduction in the occurrence of respiratory tract infections was observed in adolescents who were revaccinated with BCG compared to placebo recipients (9). Additionally, we also recently demonstrated in a randomized clinical trial (ACTIVATE) that BCG revaccination in elderly individuals can induce up to 80% reduction of the occurrence of respiratory tract infections (10).

These earlier observations have thus strengthened the hypothesis that BCG vaccine may also be effective against SARS-CoV-2 (11). This hypothesis seems to be supported by a number of observational studies that suggest a relation between BCG vaccination during childhood, low prevalence of COVID-19, and lower risk of severe COVID-19 in various countries (12, 13). Retrospective studies of BCG vaccinated individuals have also shown that BCG is safe with regard to COVID-19 (14) and seems to be related with protection against the infection or its severity (15). Based on these intriguing arguments suggesting positive effects of BCG against COVID-19, ongoing clinical prospective trials have been designed and initiated around the world to investigate the potential role of BCG vaccination (16–18). No data are yet available regarding the effectiveness of BCG vaccination in the settings of a randomized clinical trial.

## Materials and Methods

### Trial Oversight

ACTIVATE-2 (A randomized clinical trial for enhanced trained immune responses through BCG vaccination to prevent infections by COVID-19) is a prospective, double-blind, randomized, placebo-controlled, phase III clinical trial conducted in 11 departments of internal medicine in Greece. The protocol and its subsequent amendments were approved by the National Ethics Committee of Greece (approval 52/20) and by the National Organization for Medicine of Greece (approval IS 045-20) (EudraCT number 2020-002448-21; Clinicaltrials.gov NCT04414267). The trial was sponsored by the Hellenic Institute for the Study of Sepsis. The funders have no role in the design, conduct, analysis, and interpretation of data, and also decision to publish. The data lock was done on 28 April 2021. The investigators remained blind to the intervention until the results of the analysis became known.

### Patients

Study participants were informed about the study by either announcement in the media or through advertisements that were put on each of the 11 participating hospitals. Inclusion criteria were as follows: (1) adults aged 50 years or older of either sex; (2) past medical history of coronary artery disease or chronic obstructive pulmonary disease or Charlson’s comorbidity index more than 3; (3) tuberculin skin test (TST) induration diameter less than 10 mm; and (4) negative result on immunoglobulin M (IgM) or IgG serum antibodies against SARS-CoV-2.

Exclusion criteria were as follows: (1) infection by HIV; (2) any primary immunodeficiency; (3) solid organ transplantation; (4) bone marrow transplantation; (5) intake of chemotherapy or radiotherapy the last 2 months; (6) active hematological or solid tumor malignancy; (7) oral or intravenous intake of corticosteroids at a daily dose greater than or equal to 10 mg of prednisone for a period longer than the last 3 months; and (8) any anti-cytokine biological treatment. All patients or their legal representatives provided written informed consent before enrollment.

Assessment of eligibility was done after thorough study of the patient’s past history. For eligible participants, TST was done by intradermal injection of 0.1 ml of tuberculin in one forearm. Participants were asked to return after 48–72 days and, in the case of diameter of the test less than 10 mm, they were subject to a measurement of anti-SARS IgG/IgM antibodies. Antibodies were measured using the WIZ Biotech rapid measurement kit and employing single drops of blood from the forefingers. Participants negative for antibodies were allowed to be vaccinated.

### Trial Interventions

A dose of 0.1ml of sterile 0.9% sodium chloride solution or of 0.1ml of BCG vaccine was administered with an intradermal injection in the upper deltoid area. The studied BCG vaccine is a live freeze-dried vaccine derived from attenuated strain of *Mycobacterium bovis* (BCG Moscow strain 361-I) and each 0.1 ml contains 2–8 × 10^5^ colony-forming units. This was selected because it was also used in the ACTIVATE trial (10).

Following vaccination patients were asked to return at the study site four more times (study visit 2 on study days 45 ± 5; study visit 3 on study days 90 ± 5; study visit 4 on study days 135 ± 5; and study visit 5 on study days 180 ± 5). Study visits 2, 4, and 5 could also be phone visits. On each visit patients were thoroughly asked for any treatment-emergent adverse event (TEAE). Then, they were asked if they had suffered from COVID-19 diagnosed with molecular test. These cases were classified with definitive COVID-19. Then, participants were asked to provide answers to a questionnaire if they had experienced COVID-19–related symptoms. The questions were the following: presence of cough for more than 48 h; shortness of breath for more than 48 h; fever above 38°C for more than 48 h; and expectoration for more than 48 h. Patients answering positive to at least two of these were classified as possible COVID-19 cases. Patients answering positive to at least two of these and were also admitted due to their symptoms at an emergency hospital department and/or were in need of antibiotic intake and were classified as probable COVID-19 cases. Participants were also subject to a second rapid test for anti-SARS IgG/IgM antibodies on visit 3.

### Outcomes

The primary outcome was assessed on study visit 3 (study days 90 ± 5 from the date of visit 1) as the incidence of total cases of possible/probable/definitive COVID-19 the first 90 ± 5 days after vaccination. The incidence of total cases of possible/probable/definitive COVID-19, the first 135 ± 5 days after vaccination, and the first 180 ± 5 days after vaccination were the secondary study end points.

TEAEs were captured from baseline until the last patient’s evaluation. TEAEs of death, life-threatening situation, inpatient hospitalization or prolongation of existing hospitalization, and ending in persistent or significant disability/incapacity were reported as serious. All others TEAEs were graded as mild, moderate, and severe. TEAEs were also classified based on their causal relationship to the study intervention as non-related, possibly related, probably related, and definitively related.

### Statistical Analysis

Qualitative variables were expressed as percentages and 95% confidence intervals (CIs) and quantitative variables were expressed as means and standard error. Comparisons between placebo-vaccinated and BCG-vaccinated individuals were done by the Fisher’s exact test. Stepwise logistic regression analysis was done to investigate the variables associated with probable/possible/definitive COVID-19 until visit 6. The COVID-19 cases entered the equation as dependent variables and variables defined by the univariate analysis to be associated with COVID-19 as independent variables. The odds ratio (OR) and the 95% CIs were defined.

## Results

### Trial Conduct

**Figure 1:**
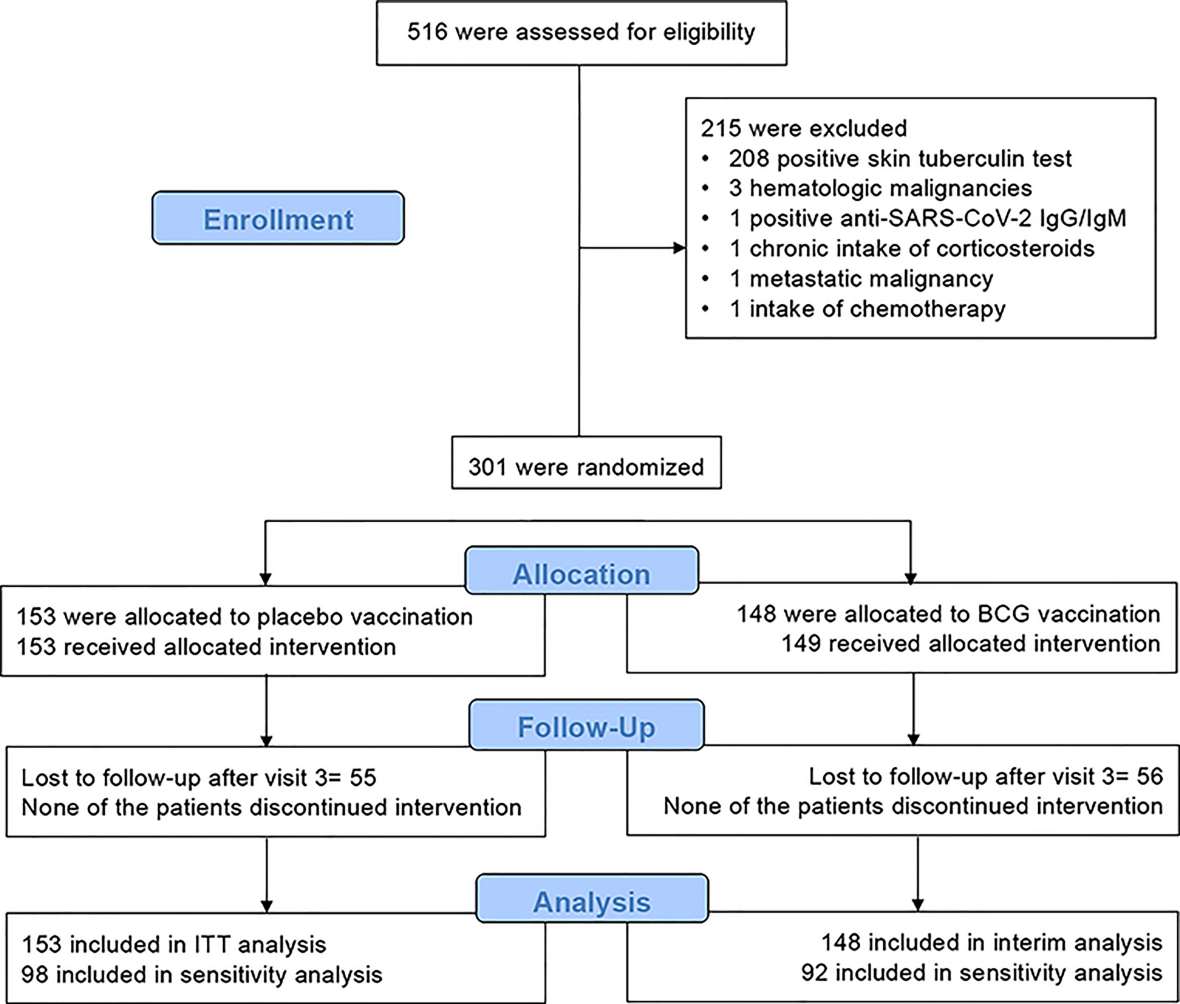
Study flow chart of ACTIVATE-2 study.

A total of 516 eligible participants were screened between June 2020 and October 2020. The first participant was enrolled on 6 June 2020 and the follow-up of the last participant was completed on 19 April 2021. No other people were willing to participate after October 2020 as the start of the regular vaccination programs for the general population has already been announced. A total of 215 individuals were excluded as they met one or more exclusion criteria (**Figure 1**). All participants were included in the 3-month analysis. The two arms had similar characteristics at baseline (**Table 1**). After the first 3 months, 55 individuals were reported as lost to follow-up in the placebo group, and 56 individuals in the BCG vaccination group; as such, 98 placebo-vaccinated individuals and 92 BCG-vaccinated individuals were included in the 6-month analysis.

**Table 1:** Demographics of enrolled patients in the placebo- and BCG-vaccinated groups.

	Placebo (n = 153)	BCG (n = 148)	P-value
Male gender, n (%)	106 (69.7)	98 (66.2)	0.538
Age (years), mean (SD)	68.7 (10.6)	68.6 (10.4)	0.912
Charlson’s comorbidity index, mean (SD)	3.86 (1.53)	3.66 (1.52)	0.112
Comorbidities, n (%)			
Coronary heart disease	25 (16.3)	17 (11.5)	0.247
Chronic heart failure	11 (7.2)	5 (3.4)	0.199
Type 2 diabetes mellitus	34 (22.2)	28 (18.9)	0.569
Vascular hypertension	49 (32.2)	38 (25.7)	0.253
Peripheral vascular disease	19 (12.4)	12 (8.1)	0.257
Chronic renal disease	5 (3.1)	1 (0.7)	0.214
Stroke	9 (5.9)	3 (2.0)	0.139
Chronic obstructive pulmonary disease	35 (22.9)	38 (25.7)	0.593
Hypothyroidism	5 (3.3)	6 (4.1)	0.767
Any surgery	36 (23.5)	39 (26.4)	0.596
Recent hospitalization	21 (13.7)	20 (13.5)	1.00

### Study End Points

**Figure 2:**
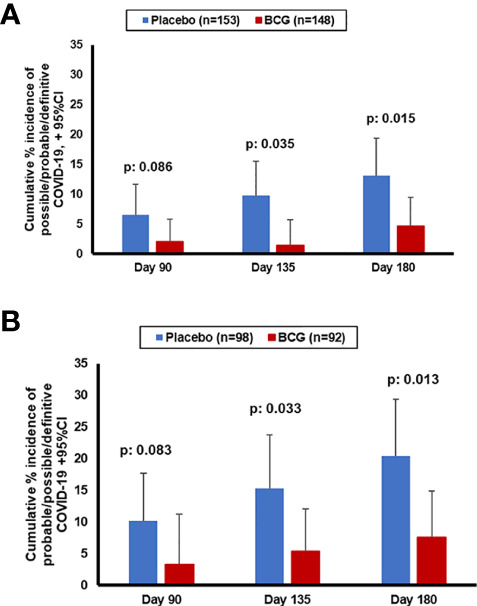
Cumulative incidence of probable/possible/definitive COVID-19 diagnosis during the study in the entire cohort **(A)** or in the patients that have completed the 180 days' follow-up **(B)**.

The primary outcome of the study was the incidence of COVID-19-possible/probable/definitive infections during the first 3-month period of follow-up after vaccination. This was a composite end point that included clinical characteristics compatible with COVID-19 and/or the laboratory diagnosis of SARS- CoV-2. The primary endpoint was met in 10 participants in the placebo group and two participants in the BCG group (p = 0.086). This may be due to the low overall incidence of COVID-19 in Greece the first 3 months after the vaccination. In contrast, 6 months after vaccination, the total number of COVID-19 diagnoses (possible/probable/definitive) was significantly lower in BCG recipients compared with the placebo recipients: OR 0.32 in multivariate analysis (95% CI 0.13–0.79, p = 0.014) (**Tables 2**, **3**; **Figure 2**). Definitive diagnosis of severe COVID-19 requiring hospitalization was present in six individuals in the placebo-vaccinated group and only two in the BCG-vaccinated group.

**Table 2:** Classification of total cases as possible, probable, or definitive by group of vaccination and need for hospitalization.

	Total patients		P-value
	Placebo (n = 153)	BCG (n = 148)	
Possible, n (%)	10 (6.5)	2 (1.4)	0.035
Probable, n (%)	3 (2.0)	3 (2.0)	1.00
Definitive, n (%)	7 (4.6)	2 (1.4)	0.173
	**Patients requiring hospitalization, n (%)**		
Possible, n (%)	0 (0)	0 (0)	1.00
Probable, n (%)	0 (0)	0 (0)	1.00
Definitive, n (%)	6 (3.9)	2 (1.4)	0.283

**Table 3:** Univariate and multivariate analysis of variables associated with the overall incidence of COVID-19 compatible symptoms and/or definitive COVID-19.

	COVID-19 positive questionnaire and/or definitive COVID-19		Univariate analysis		Multivariate analysis	
	No (n = 274)	Yes (n = 27)	OR (95%CIs)	p-value	OR (95%CIs)	p-value
BCG vaccination, n (%)	141 (51.5)	7 (25.9)	0.33 (0.14-0.81)	0.015	0.32 (0.13-0.79)	0.014
Coronary heart disease, n (%)	35 (12.8)	7 (25.9)	2.39 (0.94-6.06)	0.067	*	
Type 2 diabetes mellitus, n (%)	52 (19.0)	10 (37.0)	2.51 (1.09-5.80)	0.031	**	
Recent hospitalization, n (%)	33 (12.0)	8 (29.6)	3.08 (1.25-7.58)	0.015	3.16 (1.26-7.95)	0.014

*Variables not entering the equation because of no significance in the univariate analysis.

**Variables not entering the equation after two steps of forward step-wise analysis.

CI, confidence intervals; OR, odds ratio.

At 3 months after study intervention, 301 participants were assessed for IgG and IgM antibodies against SARS-CoV-2, in order to diagnose asymptomatic infections. Although the difference between findings of the groups did not reach statistical significance, it is worth mentioning that antibodies against SARS-CoV-2 were detected in only 1.3% (2 of 153) placebo recipients, compared to 4.7% (7 of 148) BCG recipients (p = 0.099).

### Safety

The incidence of TEAEs did not differ between the two groups. However, it needs to be reported that deaths were reported only among the placebo-vaccinated participants and that the occurrence of upper respiratory tract infections was more often in the placebo group. More participants in the BCG-vaccinated group than in the placebo group reported erythema at the injection site, as expected (**Table 4**).

**Table 4:** Serious and non-serious treatment-emergent adverse events (TEAEs).

	Placebo (n = 153)	BCG (n = 148)	p-value
Total serious TEAEs, n (%)	3 (2.0)	1 (0.7)	0.623
Deaths	3 (2.0)	0 (0)	0.248
Acute pyelonephritis	1 (0.7)	0 (0)	1.00
Upper GI tract bleeding	0 (0)	1 (0.7)	0.498
Total non-serious TEAEs	10 (6.5)	9 (6.1)	1.00
Upper respiratory tract infection	7 (4.6)	0 (0)	0.015
Vaginal candidiasis	1 (0.7)	0 (0)	1.00
Hot flashes	1 (0.7)	0 (0)	1.00
Pain/erythema at the injection site	0 (0)	5 (3.4)	0.028
Pustule/edema at the injection site	1 (0.7)	2 (1.4)	0.618
Varicella-zoster infection	0 (0)	1 (0.7)	0.492
Hyperparathyroidism	0 (0)	1 (0.7)	0.492

The events that are related to COVID-19-related symptoms are not reported in this Table.

## Discussion

In this study, we present the data of the first phase III placebo-controlled randomized clinical trial of BCG vaccination against SARS-CoV-2. Although the primary endpoint of the decrease of the incidence of possible/probable/definitive COVID-19 3-months after vaccination was not met, the trial managed to achieve the secondary endpoint and demonstrate lower incidence of possible/probable/definitive COVID-19 in a population with comorbidities 6-months after vaccination. Although the number of severe infections was small, hospitalization due to severe COVID-19 occurred in five individuals that are vaccinated with placebo but occurred in only one of the BCG-vaccinated elderly volunteers. These data argue for BCG revaccination as a potentially important tool against the COVID-19 pandemic. The protective effect of BCG vaccination was shown after adjustment for comorbidities in the multivariate analysis, making highly unlikely that comorbidities may play any confounding role in the interpretation of the results.

The ACTIVATE-2 trial was initiated based on heterologous characteristics of BCG to (i) protect against unrelated infections such as respiratory tract infections in children and adults (6–10), (ii) induce favorable outcomes against viral infections in experimental models (19), (iii) decrease viremia in an experimental human model of viral infection (20), (iv) and the demonstrated safety regarding SARS-CoV-2 infection (14). We hypothesized that BCG vaccination may be effective against both the incidence and the severity of COVID-19, and this hypothesis was confirmed by the clinical data showing 68% reduction of COVID-19 retrospective diagnoses in the vaccinated individuals. Although data on BCG vaccination of study participants were not captured, BCG vaccination was part of the national vaccination strategy at their childhood. As a consequence, the data of the current study are in line with some studies showing an association between countries that implement BCG vaccination at birth and a low prevalence of COVID-19 (12, 13), whereas studies from other countries did not reproduce these findings (21, 22). Ecological studies are also prone to bias, and thus, such arguments based on ecological analyses need to be considered with caution. On the other hand, the observation reported here, of a protective effect of BCG, is supported by the reduced COVID-19 incidence in an observational study of recent BCG vaccination (23) and by the reduced severity of this disease in children that are vaccinated with BCG (24). It should be outscored that findings repeated those of the ACTIVATE study (10) since the incidence of non-COVID upper respiratory tract infections was lower among BCG-vaccinated individuals.

Two immunological processes have been suggested to mediate non-specific protective effects of vaccines such as BCG: heterologous T-cell immunity and trained immunity (5). Heterologous T-cell responses often rely on antigenic mimicry, and recently, it has been suggested that heat shock protein 65 from BCG has sequence similarities with S-protein from SARS-CoV-2 (25). The non-specific beneficial effects of BCG vaccine and its protection against unrelated infections are attributed to epigenetic changes and metabolic remodeling of innate immune system, leading to an increased antimicrobial activity of host defense mechanisms, a process termed trained immunity (26). After a first stimulation with certain microorganisms, the innate immune system is activated and it can produce a more efficient and rapid response to secondary (and non-related) stimuli. Moreover, in *in vitro* studies, the administration of BCG resulted in a remarkably favorable immune response against viral infections such as influenza and against bacterial and fungal infections (19). Furthermore, viremia and the amount of circulating cytokines after yellow fever vaccination were lower among subjects who had been vaccinated with BCG 1 month prior to yellow fever vaccine compared to placebo-vaccinated volunteers, without differences of antibodies production between the two groups (20). The reduction in viremia correlated with myeloid-dependent trained immunity responses, rather than specific lymphocyte-dependent immune memory. The potential important role of trained immunity for the defense against SARS-CoV-2 is supported by the observation of enhanced innate immune responses post-vaccination in our recent ACTIVATE trial as well (10).

An intriguing observation was made when COVID-19 serology was investigated in order to identify the potential asymptomatic cases: only 1.3% of the placebo-vaccinated individuals were positive, whereas 4.7% of the BCG recipients showed positive SARS-CoV-2 antibodies. While the difference was not statistically significant due to the low numbers and it may be just by accident, it is tempting to speculate that this is a real observation: earlier studies have demonstrated that BCG improves serological responses to other vaccines (27, 28) and, in our sample, induced similar effects in asymptomatic COVID-19 cases. It has been demonstrated that mild or asymptomatic COVID-19 infections induce low antibody responses (29–32); in contrast, a previous BCG vaccination may improve this and indirectly induce in an increased protection against the infection in individuals asymptomatically infected with SARS-CoV-2. Future studies are warranted to explore and validate this hypothesis.

In many countries, ongoing studies are designed to investigate BCG vaccination both in children and adults. It is interesting to observe that a certain pattern starts to emerge in which trials in developing countries or countries that employed BCG vaccination programs show beneficial outcomes of BCG revaccination, whereas countries in which first BCG vaccination is administered in naïve populations of developed countries the effect seem to be less strong. In children, clinical trials in Africa have shown significant protection of BCG vaccination against mortality (6, 7) and infections (8), but such effect failed to materialize in a recent large trial in Denmark (33). Interestingly, however, in the Calmette study from Denmark, BCG vaccination induced a 30% protection of the child against infections if the mother was also BCG-vaccinated, whereas no effect was seen if the mother never received BCG (34). Similarly, BCG revaccination in South-African adolescents resulted in 73% less infections (9), whereas BCG vaccination in Greek elderly resulted in 80% less respiratory tract infections (10). While we do not have information regarding the BCG vaccination status of the volunteers in the ACTIVATE-2 trial, Greece had a policy of BCG vaccination at birth in the temporal interval when the volunteers from this trial were born. The positive data in the present study contrast the results of a recent interim analysis of the BCG-PRIME study from the Netherlands that suggested lack of significant effects of BCG vaccination (35). The existence of trained immunity by *Mycobacterium tuberculosis* is indirectly evidenced by what is known as latent tuberculosis and which is diagnosed by the positive TST or the positive interferon-gamma release assay. Cumulative data between April and May 2020 from 20 European countries suggested that the prevalence of COVID-19 was lower among the population with higher prevalence of latent tuberculosis (36). From a survey of 1,936 individuals with positive TST in Nidge, Turkey, 76 individuals had an infection by SARS-CoV-2. The severity of COVID-19 was lower among the individuals with the larger diameter of STT positivity (37).

Seven main limitations of the study need to be acknowledged: a) the small number of participants. Although the strong impact of BCG vaccination allowed to reach important conclusions regarding the effect on COVID-19, larger studies that are currently under way in different countries need to confirm the data of ACTIVATE-2. The small size of the study deters us from drawing a conclusion regarding the impact of BCG vaccine on severe forms of COVID-19, although the difference of hospitalizations between placebo and BCG groups suggests a potential protection of the vaccination; b) the lack of microbiological testing in participants with a clinical diagnosis of possible or probable COVID-19. Although we cannot exclude an alternative diagnosis is some of these patients, the context of the pandemic during tight societal restrictions makes COVID-19 diagnosis the most likely; c) the lack of measurement of biomarkers before and after vaccination that can inform on the development of trained immune responses; d) the lack of clinical data concerning the SARS-CoV-2 variants that caused the infections in this study, and future studies need to research this aspect as well; e) limited information regarding the impact of BCG on the specific immunological host defense pathways against SARS-CoV-2 (T-cell responses and specific antibodies), and additional investigations should investigate these effects in more detail; f) the use of negative anti-SARS IgG and IgM antibodies in the inclusion criteria. Seronegativity does not exclude the past history of COVID-19 and the use of one immune reactivity assay would have been more precise. However, the ease of implementation in the setting of a multicenter trial made their use attractive; and g) the lack of information on the precise BCG vaccination history of the study participants. A second BCG vaccination may act as a booster for trained immunity particularly since BCG protection is waning for people who were vaccinated before the age of 5 years (38, 39). From a similar point of view, results of the Global Burden of Disease Study report that the incidence of tuberculosis is steadily changing from 1990 to 2017; this decreased in China (40), Eastern Asia, Central Asia, Western Europe, North America, and Southern Latin America but increased in Southern Asia, Oceania, Eastern Europe, and Central Latin America (41, 42) whatever may have a major impact on the trained immunity of populations by *M. tuberculosis.*

When considering the availability of the novel specific anti–SARS-CoV-2 vaccines, one could ask why do we need trials to determine the effect of BCG against COVID-19. There are several important reasons why such studies are still very important. First, the availability of the specific vaccines is limited, especially in developing countries: as the majority of developing countries have programs of neonatal BCG vaccination, a strategy using BCG revaccination could have important impact on the COVID-19 pandemic until the specific vaccines are available. Second, it is still unclear when and at which point the control of the pandemic will be achieved in the context of the appearance of new variants of the virus, some of them show increased escape rates against the newly developed vaccines (43). The employment of trained immunity-based vaccines that boost anti-viral host defense in an antigen-independent manner could thus prove very important situations in which such viral variants become most prevailing. Third, the principle that heterologous vaccines can be employed with success in the real-life situation of a pandemic is extremely important for future pandemic preparedness: it is conceivable that a strategy employing such vaccines can be used as “bridge vaccination” for partial protection of the population even before specific vaccines are available (4). It is to be hoped that the results of the present study will encourage more research for identifying the most effective set of heterologous vaccines that can be stored, tested, and used in a future pandemic to avoid the high healthcare and societal and economic toll that can be exerted by a dangerous new pathogen.

## Data Availability Statement

The raw data supporting the conclusions of this article will be made available by the authors, without undue reservation.

## Ethics Statement

The studies involving human participants were reviewed and approved by the National Ethics Committee (approval 52/20) and by the National Organization for Medicine of Greece (approval IS 045-20) (EudraCT number 2020-002448-21; Clinicaltrial.gov NCT04414267). The patients/participants provided their written informed consent to participate in this study.

## Author Contributions

MT collected clinical data and maintained the study database, drafted the manuscript and gave final approval of the final version to be submitted. ET contributed to data analysis. drafted the manuscript and gave final approval of the final version to be submitted. KD, AK, KL, CDa, MK, MP, GA, IPa, KS, AB, KK, CDe, IPe, APan, KT, NP, EmK, ID, ES, KA, APer, GP, HM, EA, ElK, LE, VP, and APap collected clinical data, reviewed the manuscript and gave final approval of the final version to be submitted. MGN conceptualized the study, analysed the data, drafted the manuscript and gave approval of the final version to be submitted. EJGB designed the study protocol, analysed the data, drafted the manuscript and gave approval of the final version to be submitted.

## Funding

The study was funded in part by the Hellenic Institute for the Study of Sepsis and in part by Fast Grants of Emergent Ventures at the Mercatus Center, George Mason University.

## Conflict of Interest

EJGB has received honoraria from Abbott CH, InflaRx GmbH, MSD Greece, Sobi Greece and XBiotech Inc.; independent educational grants from AbbVie, Abbott, AxisShield, bioMérieux Inc, InflaRx GmbH, Sobi and XBiotech Inc; and funding from the Horizon2020 Marie-Curie Project European Sepsis Academy (granted to the National and Kapodistrian University of Athens), and the Horizon 2020 European Grants ImmunoSep and RISKinCOVID (granted to the Hellenic Institute for the Study of Sepsis). MGN was supported by an ERC Advanced Grant (#833247) and a Spinoza grant of the Netherlands Organization for Scientific Research. MN is a scientific founder of TTxD.

The remaining authors declare that the research was conducted in the absence of any commercial or financial relationships that could be construed as a potential conflict of interest.

## Publisher’s Note

All claims expressed in this article are solely those of the authors and do not necessarily represent those of their affiliated organizations, or those of the publisher, the editors and the reviewers. Any product that may be evaluated in this article, or claim that may be made by its manufacturer, is not guaranteed or endorsed by the publisher.
