# Corrigendum: ACTIVATE-2: A double-blind randomized trial of BCG vaccination against COVID-19 in individuals at risk

**DOI:** 10.3389/fimmu.2022.1018384

**Published:** 2022-08-31

**Authors:** Maria Tsilika, Esther Taks, Konstantinos Dolianitis, Antigone Kotsaki, Konstantinos Leventogiannis, Christina Damoulari, Maria Kostoula, Maria Paneta, Georgios Adamis, Ilias Papanikolaou, Kimon Stamatelopoulos, Amalia Bolanou, Konstantinos Katsaros, Christina Delavinia, Ioannis Perdios, Aggeliki Pandi, Konstantinos Tsiakos, Nektarios Proios, Emmanouela Kalogianni, Ioannis Delis, Efstathios Skliros, Karolina Akinosoglou, Aggeliki Perdikouli, Garyfallia Poulakou, Haralampos Milionis, Eva Athanassopoulou, Eleftheria Kalpaki, Leda Efstratiou, Varvara Perraki, Antonios Papadopoulos, Mihai G. Netea, Evangelos J. Giamarellos-Bourboulis

**Affiliations:** ^1^ 4^th^Department of Internal Medicine, National and Kapodistrian University of Athens, Medical School, Athens, Greece; ^2^ Department of Internal Medicine and Center for Infectious Diseases, Radboud University, Nijmegen, Netherlands; ^3^ Department of Internal Medicine, “Bodosakeio” General Hospital of Ptolemaida, Ptolemaida, Greece; ^4^ 1^st^Department of Internal Medicine, “G.Gennimatas” Athens General Hospital, Athens, Greece; ^5^ Department of Pulmonary Medicine, Aghia Eirini General Hospital of Kerkyra, Kontokali, Greece; ^6^ Department of Therapeutics, National and Kapodistrian University of Athens, Athens, Greece; ^7^ Department of Surgery, General Hospital of Argos-Unit of Nafplion, Nafplion, Greece; ^8^ 3^rd^Department of Internal Medicine, National and Kapodistrian University of Athens, Athens, Greece; ^9^ Department of Internal Medicine, General Hospital of Karditsa, Karditsa, Greece; ^10^ Nemea Health Center, Nemea, Greece; ^11^ Department of Internal Medicine, Patras University Hospital, Rio, Greece; ^12^ 1^st^Department of Internal Medicine, University Hospital of Ioannina, Ioannina, Greece; ^13^ Hellenic Institute for the Study of Sepsis, Athens, Greece; ^14^ Department of Immunology and Metabolism, Life and Medical Sciences Institute, University of Bonn, Bonn, Germany

**Keywords:** BCG, COVID-19, SARS-CoV-2, elderly vaccination, trained immunity

In the published article, there were errors in the body text.

A correction has been made to the **Abstract**. This sentence previously stated:

“These data indicate that BCG vaccination confers some protection against possible COVID-19 among patients older than 50 years with comorbidities.”

The corrected sentence appears below:

“The ACTIVATE II trial did not meet the primary endpoint of the reduction of the risk for COVID-19 3 months after BCG vaccination; however, the secondary endpoint of the reduction of the risk for COVID-19 6 months after BCG vaccination was met.”

A correction has also been made to **Results, Study End Points**, paragraph 1. This sentence previously stated:

“During these first 3 months after the vaccination the overall incidence of COVID-19 in Greece was low, and thus the number of COVID-19 diagnoses was low in both groups (10 patients in placebo vs. two participants in BCG group, p=0.086.”

The corrected sentence appears below:

“The primary endpoint was met in 10 participants in the placebo group and two participants in the BCG group (p= 0.086). This may be due to the low overall incidence of COVID-19 in Greece the first 3 months after the vaccination.”

A correction has also been made to **Discussion**, paragraph 1. This sentence previously stated:

“BCG vaccination resulted in a substantially lower incidence of possible/probable/definitive COVID-19 in an elderly population 6-months after vaccination, than in the placebo group.”

The corrected sentence appears below:

“Although the primary endpoint of the decrease of the incidence of possible/probable/definitive COVID-19 3-months after vaccination was not met, the trial managed to achieve the secondary endpoint and demonstrate lower incidence of possible/probable/definitive COVID-19 in a population with comorbidities 6-months after vaccination.”

The authors apologize for these errors and state that they do not change the scientific conclusions of the article in any way. The original article has been updated.

## Publisher’s note

All claims expressed in this article are solely those of the authors and do not necessarily represent those of their affiliated organizations, or those of the publisher, the editors and the reviewers. Any product that may be evaluated in this article, or claim that may be made by its manufacturer, is not guaranteed or endorsed by the publisher.

